# Decreasing Fertility Rate Correlates with the Chronological Increase and Geographical Variation in Incidence of Kawasaki Disease in Japan

**DOI:** 10.1371/journal.pone.0067934

**Published:** 2013-07-08

**Authors:** Yoshiro Nagao

**Affiliations:** Onoda Hospital, Minamisoma City, Fukushima, Japan; The University of Tokyo, Japan

## Abstract

**Background:**

Kawasaki disease (KD) is a common cause of acquired paediatric heart disease in developed countries. KD was first identified in the 1960s in Japan, and has been steadily increasing since it was first reported. The aetiology of KD has not been defined, but is assumed to be infection-related. The present study sought to identify the factor(s) that mediate the geographical variation and chronological increase of KD in Japan.

**Methods and Findings:**

Based upon data reported between 1979 and 2010 from all 47 prefectures in Japan, the incidence and mean patient age at the onset of KD were estimated. Using spatial and time-series analyses, incidence and mean age were regressed against climatic/socioeconomic variables. Both incidence and mean age of KD were inversely correlated with the total fertility rate (TFR; i.e., the number of children that would be born to one woman). The extrapolation of a time-series regressive model suggested that KD emerged in the 1960s because of a dramatic decrease in TFR in the 1940s through the 1950s.

**Conclusions:**

Mean patient age is an inverse surrogate for the hazard of contracting the aetiologic agent. Therefore, the observed negative correlation between mean patient age and TFR suggests that a higher TFR is associated with KD transmission. This relationship may be because a higher TFR facilitates sibling-to-sibling transmission. Additionally, the observed inverse correlation between incidence and TFR implies a paradoxical “negative” correlation between the incidence and the hazard of contracting the aetiologic agent. It was hypothesized that a decreasing TFR resulted in a reduced hazard of contracting the agent for KD, thereby increasing KD incidence.

## Introduction

Kawasaki disease (KD), first reported in the 1960s in Japan [1], mainly afflicts children younger than 5 years of age. If not promptly diagnosed and adequately treated, KD patients can develop potentially fatal cardiac sequelae [2], [3]. Although the advent of intravenous gamma-globulin therapy has dramatically improved prognosis [4], [5], KD is the most common cause of acquired paediatric heart disease in developed countries [6]. Therefore, KD patients require long-term cardiological follow up [7].

The aetiology of KD has not been identified. However, infection is believed to be involved in the pathogenesis of the disease. Children present with clinical symptoms typical of viral infection such as fever, enanthem, rash, conjunctival injection, and adenopathy [8], [9]. KD incidence displays a remarkable seasonal pattern [10]. There is a rapid geographical spread during epidemic seasons [11], and geographical and temporal clustering has been observed [12]. Additionally, there is often a short interval (<10 days) between disease onset among siblings; this delay possibly corresponds to an incubation period [13]. The age of peak incidence is 1–2 years, which is similar to common viral infections. KD rarely occurs in infants younger than 6 months, suggesting protection by passive immunity [14]. Many common infectious diseases have been proposed as the cause of KD [8], [15]–[18]. It is also possible that a novel pathogen is responsible for KD [19].

Epidemiological studies have demonstrated that the incidence of KD is highest among individuals of Asian descent and lowest among Caucasians [20], [21]. The highest incidence of KD in the world is in Japan [22]. Children of KD patients experience KD with a larger frequency than expected [23]. Additionally, multiple genetic loci have been proposed as predisposing factors for KD [24]–[30]. Therefore, genetic background seems to greatly influence susceptibility to the disease. Collectively, the general consensus is that KD most likely develops when a genetically susceptible host is infected with one or more ubiquitous infectious agents [9], [31].

Japan experienced three epidemic years of KD in 1979, 1982, and 1986 [32], after which the annual incidence of KD has consistently increased [33]. Therefore, elucidating the cause of the recent increase in KD incidence is of imminent necessity. To address this concern, the present study examined geographic and temporal variation of KD endemicity in Japan. Endemicity is often expressed as the force of infection (i.e., the hazard of contracting the aetiologic agent, expressed as the probability of a naive individual becoming infected in a unit of time). The hazard of contracting the aetiologic agent may not reliably be represented by incidence. The relationship between incidence and the hazard of contracting the aetiologic agent can be influenced, for example, by endemic stability [34].

In contrast to incidence, the mean age of patients (A) is assumed to be an inverse surrogate of the force of infection (or the hazard of contracting the aetiologic agent) (λ) for an infectious agent that can confer life-long sterile immunity [35], as in:
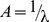
(1)


This approximation may not be applicable to a pathogen that does not confer sterile lifelong immunity, or to a disease that is caused by multiple pathogens. However, in an environment in which risk of infection with the pathogen(s) is high, it is expected that an individual would contract their first infection at an early age. Therefore, the negative correlation between the mean patient age and the hazard of contracting the aetiologic agent may still hold true.

Incidence and mean patient age can be affected by a variety of factors including climate, availability of health care facilities/providers, genetic susceptibility, socioeconomics, and demographic parameters. The impact that birthrate exerts on spatiotemporal behaviour has been an important topic of investigation in infectious disease epidemiology [36]–[38]. The present study examined the climatic, socioeconomic, and demographic factors that may have influenced the observed geographic variation and temporal change of these epidemiologic parameters in Japan.

## Methods

### KD Data

**Figure 1:**
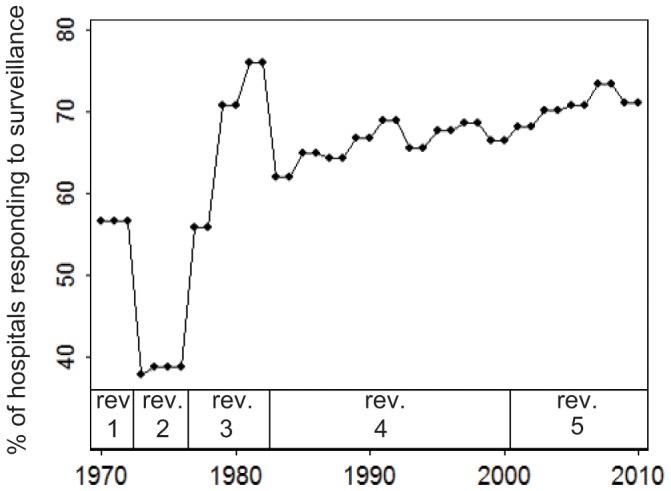
Percentage of hospitals that responded to national surveillance and the diagnostic guideline revisions. Definitions of revisions 3, 4 and 5 are available in Text S1.

The nationwide KD surveillance in Japan, which began in 1970, is currently conducted biennially. Questionnaires are sent to all hospitals with 100 or more beds and a paediatric department and to hospitals that specialize in paediatrics [39]. When diagnostic guidelines for KD are revised, they are sent to all of these hospitals. Prefecture-level data has been available since 1977 for all 47 prefectures in Japan. Since 1979, the reporting rate has consistently surpassed 60% (Figure 1). In addition, the climatic data that were used in the present study has been available since 1979. Therefore, the present study used KD case data that has been collected since 1979. The diagnostic criteria used during the study period (revisions 3, 4, and 5, Figure 1) were stable (Text S1) [40].

### Estimation of Incidence and Mean Patient Age of KD

The annual incidence of KD was estimated as the total number of KD cases occurring at any age, over the size of population aged between 0 and 4 years (unit:/100,000 children/year). In addition, age-adjusted incidence was estimated using the direct method and Japan’s age-population structure in 2000 as the standard structure. From the prefecture-level, KD cases were stratified into 19 age classes: 0–2 months (m), 3–5 m, 6–8 m, 9–11 m, 12–14 m, 15–17 m, 18–20 m, 21–23 m, 2 years (y) −2 y 5 m, 2 y 6 m–2 y 11 m, 3 y–3 y 5 m, 3 y 6 m–3y 11 m, 4 y–4 y 11 m, 5 y–5 y 11 m, 6 y–6 y 11 m, 7 y–7 y 11 m, 8 y–8 y 11 m, 9 y–9 y 11 m and ≥10 y. Mean patient age was estimated with the following equation [41], [42]:
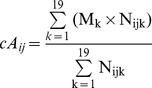
(2)where cA*_ij_* denotes the crude mean patient age at the i^th^ prefecture [i = 1–47] in year j; M_k_ represents the mid-point for k^th^ age class [k = 1–19]; N_ijk_ represents the number of patients in this age class. The demographic structure may affect the mean patient age estimated by eq. 2. For example, in a prefecture where the proportions of infants and small children are extremely high, the proportion of patients in these age groups may also be larger. As a result, the mean patient age in such a prefecture would be lower simply because of its demographic structure. To adjust for this effect of demographic structure, mean patient age was adjusted [42], as in:
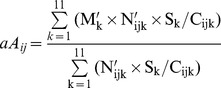
(3)where aA_ij_ represents adjusted mean patient age. S_k_ represents the proportion of the population in the k^th^ age class in the standard population structure (i.e., Japan’s population structure in 2000), and C_ijk_ represents the proportion of the population in the k^th^ age class in the i^th^ prefecture, year j. Because the minimal resolution of age class in the census data is 1 year, the original 19 age classes (M, N) were aggregated into 11 age classes (M’, N’): <1 y, 1 y–1 y 11 m, 2 y–2 y 11 m,., 9 y–9 y 11 m, and ≥10 y. KD patients were rarely more than 10 years of age. The midpoint age for this oldest class was set to 10.5 years or 15 years in two sensitivity analyses. Because the results of the statistical analyses did not change (data not shown), the results based upon the former midpoint age are presented.

Climatic/socioeconomic variables were obtained to explore the factors that contribute to the spatial and temporal variation in KD incidence and mean patient age.

### Climatic Data

A monthly summary of climatic data recorded since 1979 was obtained from the University Corporation of Atmospheric Research [43]. Temperature and precipitation were the only variables reported for 1979–1986. Additional variables were reported after 1986. To be consistent, the present study included only temperature and precipitation. Using geographically coded population data [44] and a geographic information system software ArcGIS 10 (CA, USA), the present study selected 249 weather stations in Japan that were located in cities, towns, or villages with a population of at least 10,000 people. Among these stations, 123 stations that reported data for at least 90% of the months between 1979 and 2010 were employed. The data reported from these stations were averaged for the corresponding prefecture for the following climatic variables: mean temperature (°C) and rainfall (mm/month).

### Socioeconomic/Demographic Variables

Five socioeconomic/demographic variables (Table 1) were surveyed by multiple government departments in Japan, and were recorded at least earlier than 1979 [45]–[48]: number of physicians per 100,000 individuals (variable “physician”), population density (/km^2^), percentage of population aged 65 years or older (“aged population”), percentage of students who proceeded to education beyond obligatory education (“higher education”), and total fertility rate (TFR). TFR is a measure of the average number of children that would be born to a woman over her lifetime if she were to experience the present age-specific fertility rates through her lifetime, and if she were to survive to the end of her reproductive life.

**Table 1:** Definition and availability of explanatory variables at the prefectural level.

Variable	Unit	Interval	Oldest prefectural data available	Source
Mean temperature	°C	annual	1979	[43]
Rainfall	mm/month	annual	1979	[43]
Physician	/100,000 individuals	biennial	1986	[45]
Population density	/km^2^	annual	1975	[46]
Aged population	%	annual	1975	[46]
Higher education	%	annual	1975	[47]
TFR	dimensionless	annual	1975	[48]

### Regression Analysis

The correlation between dependent variables (incidence and mean patient age) and explanatory variables (two climatic and five socioeconomic variables) was quantified. Before regression analyses, the dependent variables were transformed into a normal distribution using the following equation:
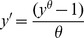
(4)where *y* (the dependent variable before transformation) was normalized to *y’*, while θ was estimated using the Box-Cox method [49].

Three regression methodologies were adopted: conventional regression, spatial regression, and time-series regression analyses. These regressions were performed as univariate and multivariate analyses. All independent variables were initially included in the multivariate analysis; the non-significant variables (P≥0.05) were eliminated in a stepwise manner. Stata 11.1 (TX, USA) was used for statistical analyses.

### Conventional Regression Analysis

Strictly speaking, the inverse relationship between the force of infection and mean age (eq. 1) holds true only for a long-term equilibrium. Diverse factors (e.g., mixing pattern, school-term, and seasonality) generate seasonal oscillations in incidence and mean patient age for infectious diseases [50] and KD [51]. To maximally circumvent the interference by these seasonal fluctuations, the age-stratified number of cases was pooled through the study period of 1979–2010: 
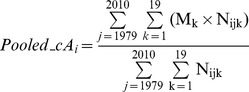
(2)

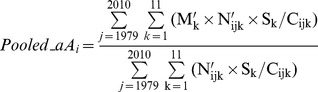
(3)


Incidence over the 0–4 year old population was estimated using the age-stratified population data averaged for the period and was completely correlated with the age-adjusted incidence (Figure S1). Therefore, only the incidence over the 0–4 year old population was used for the subsequent analyses. The explanatory variables were averaged for each period. The normalized dependent variables were regressed against explanatory variables using conventional linear regression techniques.

### Spatial Regression Analysis

The prefectures were not independent samples; prefectures located next to each other tended to exhibit similar values. Spatial regression analysis was employed to adjust for this spatial autocorrelation [52], [53], as in:
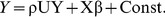
(5)where ***Y*** denotes the dependent variable vector, ***X*** represents the independent variable matrix, ρ represents the spatial autoregressive parameter, **β** denotes the regression coefficient vector, and ***U*** represents the spatial adjacency matrix. Each element of ***U*** assumed a value of “1” if two prefectures were geographically adjacent or were connected by a bridge/tunnel, or “0” if prefectures were not adjacent or connected. Three bridges were constructed (1988, 1998, and 1999) to connect prefectures. An alternative spatial adjacency matrix that did not consider these bridges did not affect the results (data not shown).

### Time-series Analysis

The conventional and spatial regression analyses did not consider the effect of time lag. To consider time lag, independent variables were transformed:
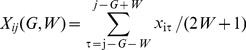
(6)where *X*_ij_ (G, W) was an independent variable that was regressed against a dependent variable recorded in i^th^ prefecture at year j. *G* represented the lag and *W* defined the smoothing radius within which the independent variables were averaged. For example, *X_i,j = 2010_* (*G = *20, *W = *5) was an average of the values recorded between 1985 ( = 2010–20–5) and 1995 ( = 2010–20+5). For each univariate pair of dependent and explanatory variables, the optimal combination of *G* and *W* was selected to generate the largest overall R^2^ using the dependent variable recorded between 2000 and 2010. A biennially surveyed explanatory variable–physician–was linearly interpolated to the year for which the value was not available.

A random-effect first-order autoregressive model was employed to adjust for serial autocorrelation:
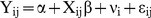
(7)where *Y_ij_* was the dependent variable and ***X*** *_ij_* was the independent variable that accounted for the time lag for the i^th^ prefecture, year j. *α* was a constant term, ***β*** the regression coefficient, and ν*_i_* was a random effect term that was distributed independently and identically. To express serial autocorrelation, ε_ij_ was assumed to be correlated over time:
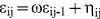
(8)where η_ij_ is a noise term.

## Results

### Determinant for Geographic Variation of Incidence and Mean Age of KD

**Figure 2:**
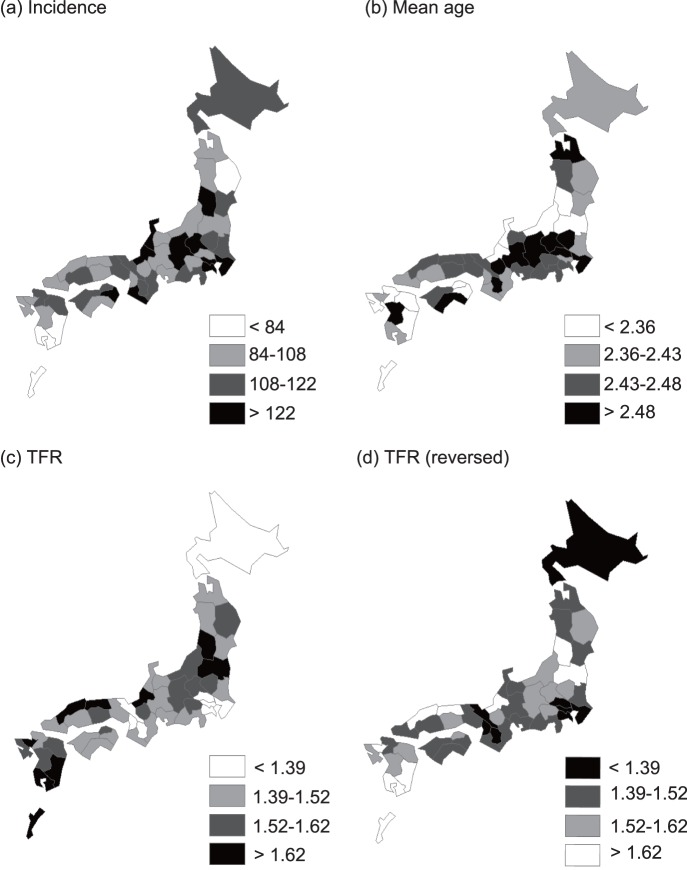
Total fertility rate (TFR), incidence, and mean age of Kawasaki disease, 1979–2010. Prefectures were classified based upon annual incidence (/100,000 children between 0 and 4 years) (a), crude mean patient age (years) (b), and TFR (c). TFR was also presented in reverse order (d). The similarity between (a) and (d), and that between (b) and (d) implied negative correlations between incidence and TFR, and between mean patient age and TFR, respectively. The cut-off values were selected by natural breaks classification algorithm. Only the main islands are presented.

Normalized incidence was regressed against climatic/socioeconomic variables, aggregated in the study period (Table 2). In both conventional and spatial regressions, only the TFR consistently demonstrated a statistically significant (*P*<0.05) contribution to the incidence of KD. Regression coefficients derived from normalization are difficult to interpret intuitively; therefore, results from non-normalized dependent variables are presented (Table S1, Table S2, Table S3 and Table S4) to demonstrate that non-normalization did not qualitatively affect the results. Table 3 shows the results from the regression of normalized mean patient age (estimated by eqs. 2′ and 3′) against climatic/socioeconomic variables. In conventional multivariate analysis, only the TFR showed a statistically significant contribution to both crude and adjusted mean patient ages of KD. In the spatial multivariate analysis, only the TFR remained as a statistically significant contributor to adjusted mean patient age. Collectively, the TFR was inversely correlated with the incidence of KD and with the mean patient age of KD (Figure 2).

**Table 2:** Regressive models to explain incidence of Kawasaki disease over 0–4 year population, averaged between 1979 and 2010.

Variables	θ for Box Cox’s transformation = 1.8
**Univariate regression**	**(n = 47)**
Mean temperature	−45 (*P* = 0.377)
R^2^	0.017
Rainfall	−1.8 (*P* = 0.675)
R^2^	0.0039
Physician	2.5 (*P* = 0.504)
R^2^	0.010
Population density	0.10 (*P* = 0.335)
R^2^	0.021
Aged population	−22 (*P* = 0.628)
R^2^	0.0053
Higher education	152 (*P* = 0.139)
R^2^	0.048
TFR	−2,931 (*P* = 0.001)
R^2^	0.21
**Conventional multivariate regression** *	**(n = 47)**
TFR	−2,931 (*P* = 0.001)
R^2^	0.21
**Spatial multivariate regression** *	**(n = 47)**
TFR	−2,828 (*P* = 0.001)
* Ρ*	0.0092
R^2^	0.21

* : Only TFR remained as the statistically significant contributor to the multivariate model in both conventional and spatial regressions.

**Table 3:** Regressive models to explain mean age of Kawasaki disease patients pooled between 1979 and 2010.

	Crude mean age	Adjusted mean age
Variables	θ for Box Cox’s transformation = 12	θ for Box Cox’s transformation = 8.8
**Univariate regression**	**(n = 47)**	**(n = 47)**
Mean temperature	−96 (*P* = 0.262)	−0.22 (*P* = 0.304)
R^2^	0.028	0.024
Rainfall	−3.9 (*P* = 0.592)	−0.014 (*P* = 0.440)
R^2^	0.0064	0.013
Physician	−8.3 (*P* = 0.175)	−0.013 (*P* = 0.394)
R^2^	0.041	0.016
Population density	−0.013 (*P* = 0.943)	0.0010 (*P* = 0.016)
R^2^	0.0001	0.12
Aged population	−44 (*P* = 0.560)	−0.39 (*P* = 0.029)
R^2^	0.0076	0.10
Higher education	−64 (*P* = 0.715)	−0.29 (*P* = 0.501)
R^2^	0.0030	0.010
TFR	−3,289 (*P* = 0.039)	−11 (*P* = 0.003)
R^2^	0.091	0.19
**Conventional multivariate regression** *		
TFR	−3,289 (*P* = 0.039)	−11 (*P* = 0.003)
R^2^	0.091	0.19
**Spatial multivariate regression** †		
TFR		−8.4 (*P* = 0.009)
* Ρ*		0.065
R^2^		0.36

* Only TFR remained as the statistically significant contributor to the conventional multivariate models for both crude and adjusted mean patient age.

† In spatial regression, no variable remained as a significant contributor to crude mean age and only TFR remained as the significant contributor to adjusted mean age.

### Temporal Shift in Epidemiological Parameters and Shift Determinants

**Figure 3:**
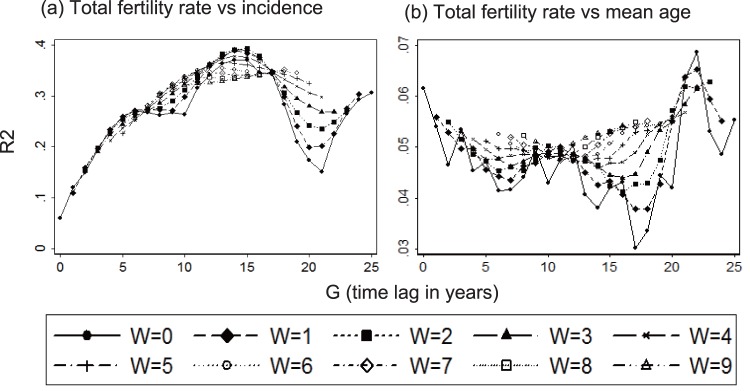
Fitness (R^2^) of the time-series regression model, time lag (*G*), and smoothing radius (*W*). Incidence (a), and crude mean patient age (b) of Kawasaki disease were regressed against the TFR estimated for a specific combination of *G* (time lag) and *W* (smoothing radius). The *G* and *W* are defined in eq. 6.

Using time series regression analysis, normalized incidence and mean patient age, recorded between 2000 and 2010, were regressed against climatic/socioeconomic variables (eqs. 7 and 8). A condition of *W*>0 was assumed, because, at *W* = 0, relationships between the fitness of the regression (R^2^) and *G* were unstable for many explanatory variables (Figure 3 and Figure S2). Multivariate time-series regression analyses (Table 4) demonstrated that KD incidence was correlated with the TFR (*G* = 15, *W* = 2) and an aged population (*G* = 13, *W* = 1). Table 5 showed that crude and adjusted mean patient ages were correlated with the TFR (*G* = 22, *W* = 1 for crude mean age; *G* = 20, *W* = 1 for adjusted mean age) and physician (*G* = 2, *W* = 1). To test the validity of these results, the multivariate time-series regression analysis was applied to the entire dataset (i.e., 1979–2010). Qualitatively similar results were obtained (Table 4 and Table 5).

**Table 4:** Time-series random-effect regressive models to explain Kawasaki disease incidence over 0–4-year old population.

			Incidence (θ = 0.34)
**Univariate analysis**			**2000–2010**
	***G***	***W***	**(n = 517)**
Mean temperature	20	1	−0.049 (*P* = 0.423)
R^2^			0.011
Rainfall	20	1	−0.0078 (*P* = 0.041)
R^2^			0.013
Physician	19	1	0.039 (*P*<0.001)
R^2^			0.22
Population density	21	1	0.00025 (*P* = 0.105)
R^2^			0.017
Aged population	13	1	0.46 (*P*<0.001)
R^2^			0.069
Higher education	13	1	0.66 (*P*<0.001)
R^2^			0.062
TFR	15	2	−8.0 (*P*<0.001)
R^2^			0.39
**Multivariate analysis**			**2000–2010**
	***G***	***W***	**(n = 517)**
Aged population	13	1	0.17 (*P<*0.001)
TFR	15	2	−6.1 (*P*<0.001)
R^2^			0.43
**Multivariate analysis**			**1979–2010**
	***G***	***W***	**(n = 893)**
Aged population	13	1	0.30 (*P<*0.001)
TFR	15	2	−6.1 (*P*<0.001)
R^2^			0.61

**Table 5:** Time-series random-effect regressive models to explain mean age of KD.

			Crude mean age (θ = 0.53)				Adjusted mean age (θ = 0.41)
**Univariate analysis**			**2000–2010**				**2000–2010**
	***G***	***W***	**(n = 517)**		***G***	***W***	**(n = 517)**
Mean temperature	20	1	−0.015 (*P*<0.001)		14	1	−0.0078 (*P* = 0.031)
R^2^			0.059				0.015
Rainfall	19	1	−0.00069 (*P* = 0.034)		6	2	−0.00066 (*P* = 0.014)
R^2^			0.014				0.017
Physician	2	1	−0.00056 (*P* = 0.038)		2	1	−0.00060 (*P* = 0.010)
R^2^			0.0239				0.022
Population density	3	1	4.1×10^−6^ (*P* = 0.675)		1	1	0.000022 (*P* = 0.003)
R^2^			0.0009				0.035
Aged population	22	1	−0.0020 (*P* = 0.657)		7	1	−0.0079 (*P*<0.001)
R^2^			0.0036				0.048
Higher education	11	1	0.016 (P = 0.011)		2	1	−0.0078 (*P* = 0.279)
R^2^			0.021				0.0024
TFR	22	1	−0.25 (*P*<0.001)		20	1	−0.22 (*P*<0.001)
R^2^			0.0653				0.063
**Multivariate analysis**			**2000–2010**				**2000–2010**
	***G***	***W***	**(n = 517)**		***G***	***W***	**(n = 517)**
Physician	2	1	−0.0010 (*P* = 0.001)		2	1	−0.00052 (*P* = 0.013)
Aged population					7	1	−0.0059 (*P* = 0.006)
Higher education	11	1	0.013 (*P* = 0.013)				
TFR	22	1	−0.29 (*P*<0.001)		20	1	−0.27 (*P*<0.001)
R^2^			0.13				0.12
**Multivariate analysis**			**1979–2010**				**1979–2010**
	***G***	***W***	**(n = 611)**		***G***	***W***	**(n = 705)**
Physician	2	1	−0.0010 (*P*<0.001)		2	1	−0.00059 (*P* = 0.006)
Aged population					7	1	−0.0043 (*P* = 0.029)
Higher education	11	1	0.013 (*P* = 0.007)				
TFR	22	1	−0.37 (*P*<0.001)		20	1	−0.32 (*P*<0.001)
R^2^			0.17				0.13

### Reconstruction of KD Incidence and Mean Patient Age

**Figure 4:**
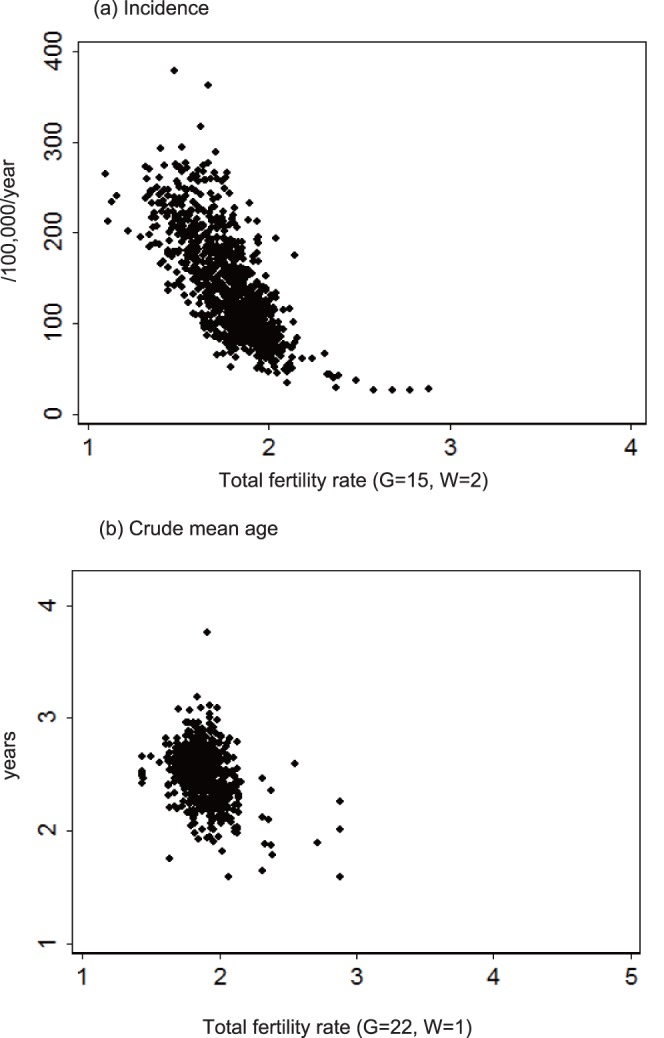
The relationship of Kawasaki disease incidence and mean patient age to Total fertility rate (TFR). KD incidence (/100,000 children between 0 and 4 years) (a), and crude mean patient age (b) were plotted against the TFR with the time lag (*G*) and smoothing radius (*W*) that provided the largest R^2^. Each dot represents an annual record from a prefecture.

**Figure 5:**
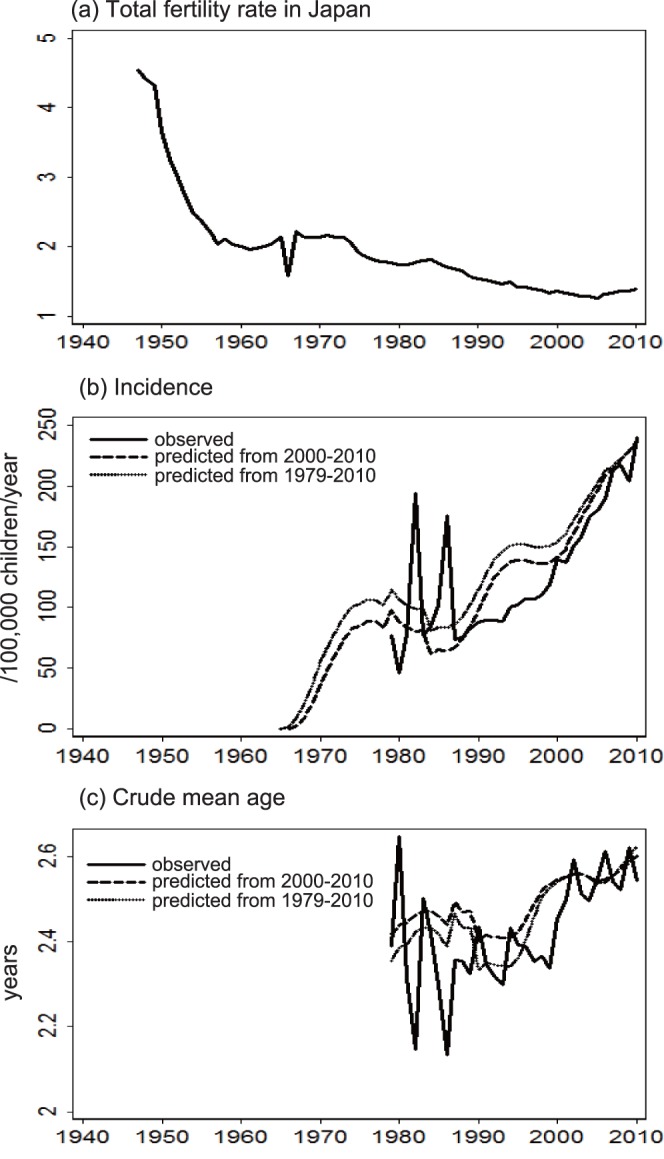
Temporal change in Total Fertility Rate (TFR) and Kawasaki disease incidence and mean patient age. KD incidence in children of 0–4 years (b) and crude mean patient age (c) were reconstructed based on the TFR in Japan (a). The reconstruction used a univariate time-series model based on Kawasaki disease data recorded either between 2000 and 2010 or between 1979 and 2010.

Collectively, these results (Table 2, Table 3, Table 4, and Table 5) suggested that the TFR was the critical parameter that affected both KD incidence and the mean patient age (Figure 4). Therefore, the incidence was reconstructed by incorporating the nation-level TFR (Figure 5a) into the univariate time-series model (Table 4). The reconstructed incidence (Figure 5b) captured the overall upward trend of incidence although the epidemic years were not reproduced. The reconstruction of incidence was further extrapolated in a retrograde manner, which suggested that KD emerged in the 1960s (Figure 5b), following a dramatic fall in the TFR in 1940–1950s (Figure 5a). The predicted mean patient age (Figure 5c) reproduced the general increasing trend of the observed mean age, but failed to reproduce the turbulences in the epidemic years.

### Age-stratified KD Data

**Figure 6:**
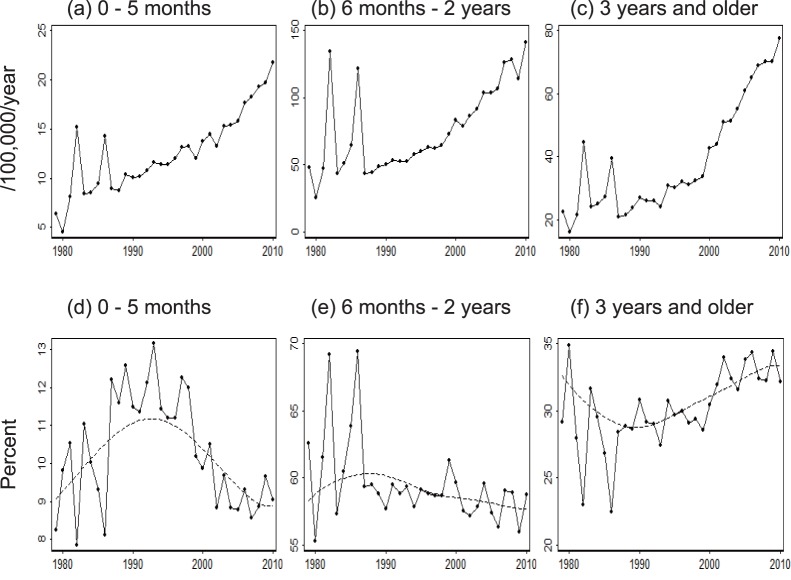
The proportion of age-stratified number of Kawasaki disease cases. Number of Kawasaki disease cases in each age category was divided by the population of children between 0 and 4 years (a, b, c). The percentage of patients in each age category was smoothed using the Lowess spline procedure (dashed line) (d, e, f).

To explore the mechanism that could explain the link between the decreasing TFR and the increasing KD incidence, the available KD data was examined in detail. The number of KD cases increased over three decades for all 19 age-classes (Figure S3). This increase was most notable in infants between 0–2 months of age, from 0.83/100,000 children 0–4 years of age in 1979 to 4.9/100,000 children in 2010. Proportional expression revealed heterogeneity between the age-classes (Figure S4). The age classes were re-classified into 3 categories: 0–5 months, 6 months–2 years, 3 years and above (Figure 6). The number of KD cases increased in all three categories (Figure 6 a–c). However, the first half of the three decades was dominated by a proportional increase in KD in infants 0–5 months, while the latter half was characterized by a proportional increase in children 3 years or older (Figure 6 d–f).

## Discussion

Much attention has been paid to risk factors for KD incidence, in terms of climate [54]–[57] and socioeconomic status [54], [55], [58]. While the climatic risk factors identified by these studies were inconsistent [54]–[57], two studies suggested that higher socioeconomic status may be a positive risk factor [54], [58]. The present study attempted to identify factor(s) that contribute to the changing epidemiology of KD in Japan. It was demonstrated that the TFR was negatively correlated with both KD incidence and mean patient age, even after adjusting for climatic/socioeconomic conditions. These correlations were statistically significant across both space and time.

The negative correlation between the TFR and the mean patient age suggested that a higher TFR may promote the hazard of contracting the aetiologic agent for KD. This finding can be explained by the fact that a large total fertility rate indicates the birth of many children in a family and in the same generation; this would most likely facilitate sibling-to-sibling and friend-to-friend transmission. Indeed, presence of a sibling has been reported as an important risk factor for paediatric infectious diseases [59]–[62].

As a result, the observed negative correlation between the TFR and KD incidence may imply an inverse association between the hazard of contracting the aetiologic agent and KD incidence. This paradoxical finding is reminiscent of endemic stability [34]. Endemic stability is a state characterized by a stably low incidence of symptomatic illness at a high force of infection. This peculiar phenomenon occurs in a pathogen that manifests symptomatic illness more frequently in older individuals than in younger individuals. Under a very high force of infection, illnesses from such a pathogen would be masked because infections occur mainly in younger children. To explore the possibility of endemic stability, age-stratified KD data was examined. The first half of the study period was dominated by an increased percentage of KD cases in children 0–5 months of age. This finding suggested that endemic stability was an unlikely cause of the increase in KD incidence during this period. Instead, the increase in incidence during the first period may have been driven by a decrease in passive immunity. This “inefficient passive immunity” hypothesis is elaborated in Text S2, Figure S5, and Figure S6. In brief, infection/boosting of a female by her sibling(s) may elevate the antibody titre in this female who would subsequently transfer a large amount of protective maternal antibody to her baby. An alternative, but not exclusive, hypothesis is that an infected child may boost its mother, who would transfer elevated levels of protective antibodies to subsequent offspring. The first hypothesis can be tested with an epidemiological study that compares the number of siblings among the mothers of KD patients and mothers of control subjects. The second hypothesis can be tested by comparing the number of older siblings between KD patients and control subjects. Comparison of the incidence of KD between children based upon birth order would be useful as a test of the second hypothesis.

Another explanation for the proportional increase in 0–5 month-old children during the period from the late 1980’s to late 1990’s is that this may have been the most susceptible age group that remained after the KD epidemics, which mostly affected the older age groups. Mathematical modeling methodology could be used to compare this hypothesis with the inefficient immunity hypothesis.

In the present study, climatic factors did not exhibit significant impact on the epidemiology of KD. This result may seem inconsistent with the sharp peak of KD incidence which usually appears in January in Japan. However, between 2000 and 2010, the number of KD cases which occurred in January constituted only 11% of the total number of cases. The numbers of cases which occurred in December-February period, and those which occurred in June-August period were comparable (i.e., 29% and 26%, respectively). These findings imply that the epidemic with KD in Japan may have weak seasonality. Therefore, the relationship between the mean patient age and the hazard of contracting the aetiologic agent (or force of infection) can be explained by the traditional mathematical model (i.e., eq. 1), and not by the recent model proposed by Pitzer and Lipsitch [50].

The results of the present study should be interpreted with caution because mean patient age is an inverse of the force of infection when birthrate is equal to mortality (i.e., for a stable population). When population size changes, mean patient age is affected not only by force of infection but by population growth rate and mortality rate [63]. Even for a growing population, however, mean patient age is a good estimator of the inverse of the force of infection [64]. A recent study predicted that the mean patient age is affected by the force of infection much more than by the population growth rate (page 220 in ref. [65]). Therefore, the statistically significant negative correlation between the mean patient age of KD and TFR suggests that TFR is actually correlated with the hazard of contracting the aetiologic agent of KD.

Another limitation of the present study is the small sample size (i.e., 47 prefectures), which made the analysis vulnerable to confounding factors (i.e., ecological fallacy). Therefore, the aim of this study is not to present a definitive conclusion but to propose a hypothesis to facilitate future studies.

The results of the present study suggest that the increasing incidence of KD in Japan may be an example of an “epidemiological disequilibrium”, which has resulted from demographic disequilibrium [66]. The TFR has been declining not only in Japan, but in many countries/regions [67]. Therefore, elucidating the causal link between this demographic parameter and KD using epidemiological, geographical, and simulation studies is of paramount importance.

## Supporting Information

Figure S1 Correlation between age-adjusted incidence and incidence over 0–4 year old population.
(TIF)

Figure S2 Fitness of time-series regression compared across G and W.
(TIF)

Figure S3 Number of KD in 19 age classes.
(TIF)

Figure S4 Percentage of KD patients in 19 age classes.
(TIF)

Figure S5 Hypothesis: decreasing total fertility rate may reduce the titre of maternal antibody, thereby increasing the incidence of KD.
(TIF)

Figure S6 Alternative hypotheses. (a) Total fetility rate may affect the number of antibody species which are passively transferred. (b) Total fertility rate may affect the efficiency of passive immunity through children-to-mother interaction.
(TIF)

Table S1 Regression analyses from Table 2 (main text) was applied to non-normalized incidence of KD over 0–4 year old population (n = 47).
(DOC)

Table S2 Regressive analyses from Table 3 (main text) was applied to non-normalized mean age of KD patients (n = 47).
(DOC)

Table S3 Regression analyses from Table 4 (main text) was applied to non-normalized KD incidence.
(DOC)

Table S4 Regression analyses from Table 5 (main text) was applied to non-normalized mean patient age.
(DOC)

Text S1 Definition of Kawasaki disease.
(DOC)

Text S2 Hypotheses to explain increasing incidence of KD based upon decreasing total fertility rate.
(DOC)
