# Supplementary figures and images for: Decreasing Fertility Rate Correlates with the Chronological Increase and Geographical Variation in Incidence of Kawasaki Disease in Japan
Source: PLoS One. 2013 Jul 8;8(7):e67934. doi: 10.1371/journal.pone.0067934 (PMC3704585; doi:10.1371/journal.pone.0067934)

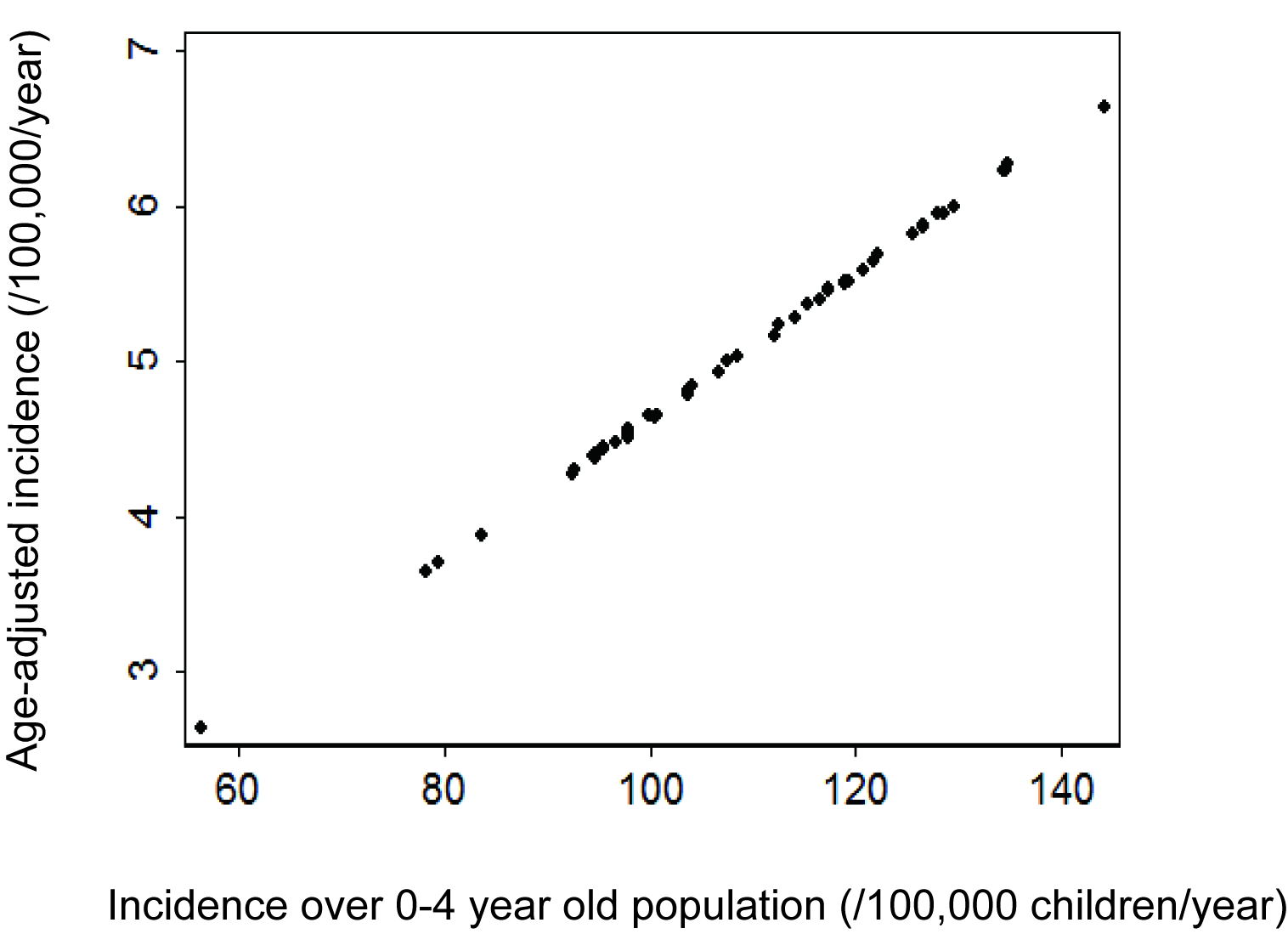

Supplement: Figure S1 — Correlation between age-adjusted incidence and incidence over 0–4 year old population. (TIF) [file pone.0067934.s001.tif]

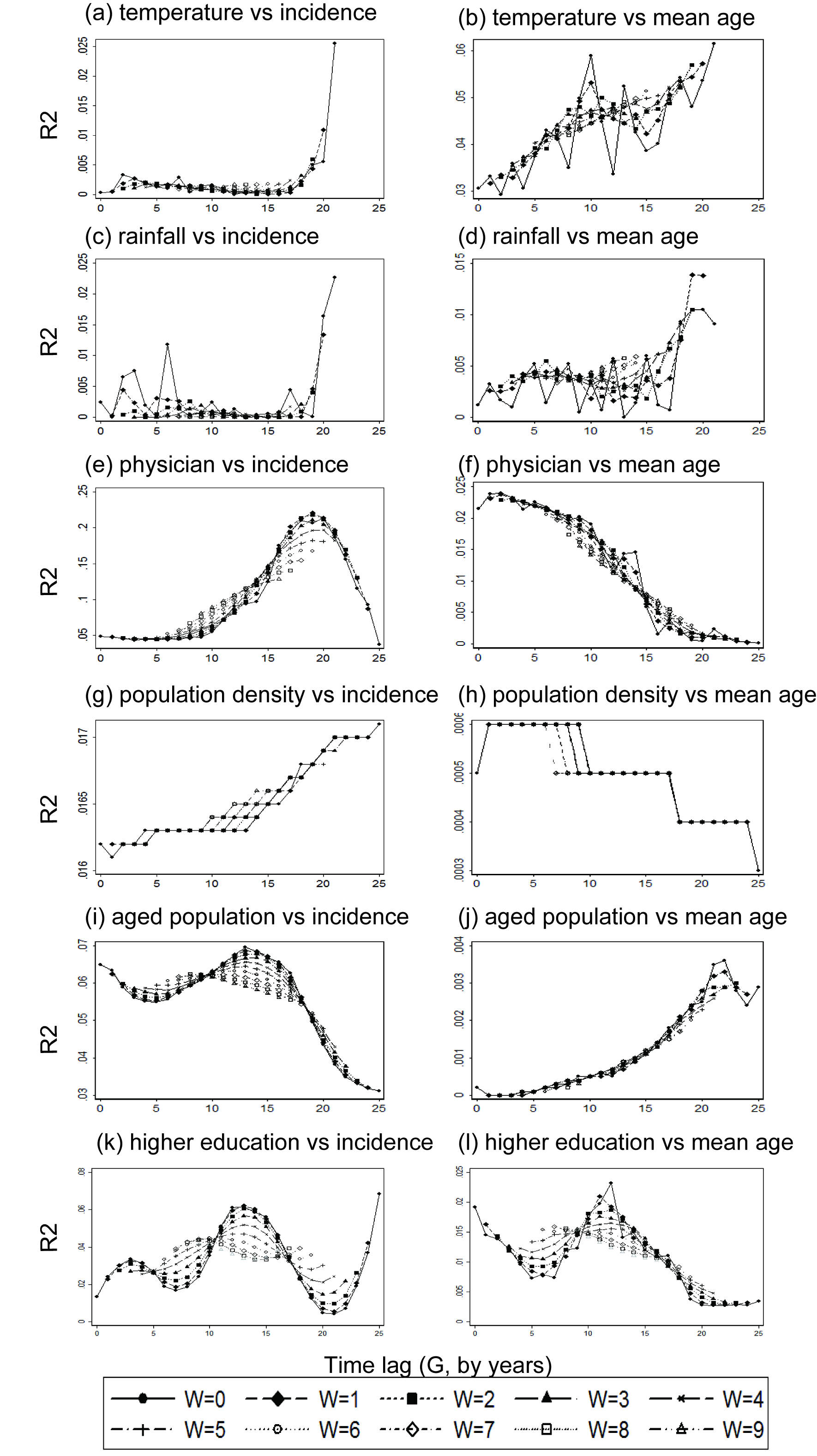

Supplement: Figure S2 — Fitness of time-series regression compared across G and W. (TIF) [file pone.0067934.s002.tif]

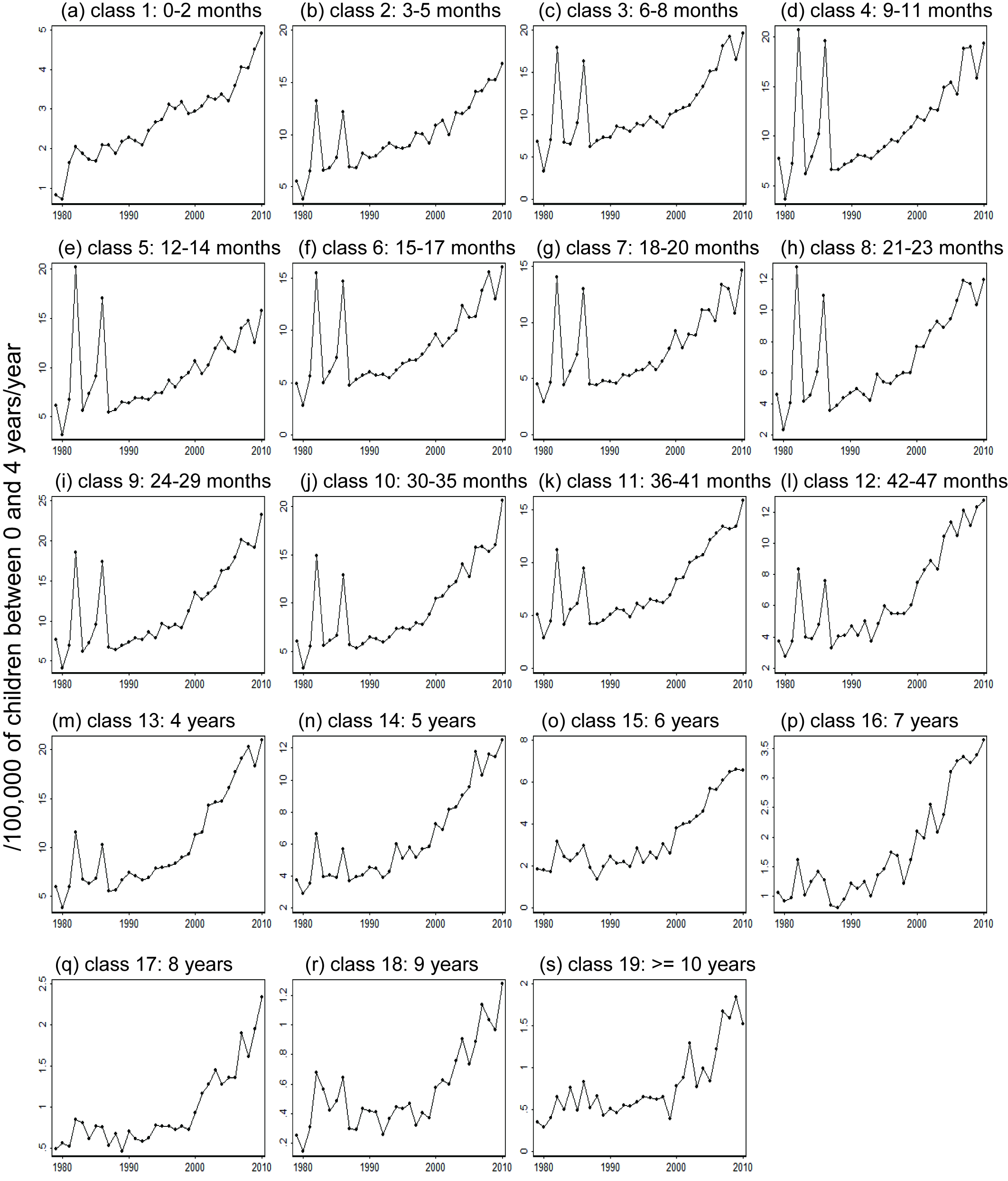

Supplement: Figure S3 — Number of KD in 19 age classes. (TIF) [file pone.0067934.s003.tif]

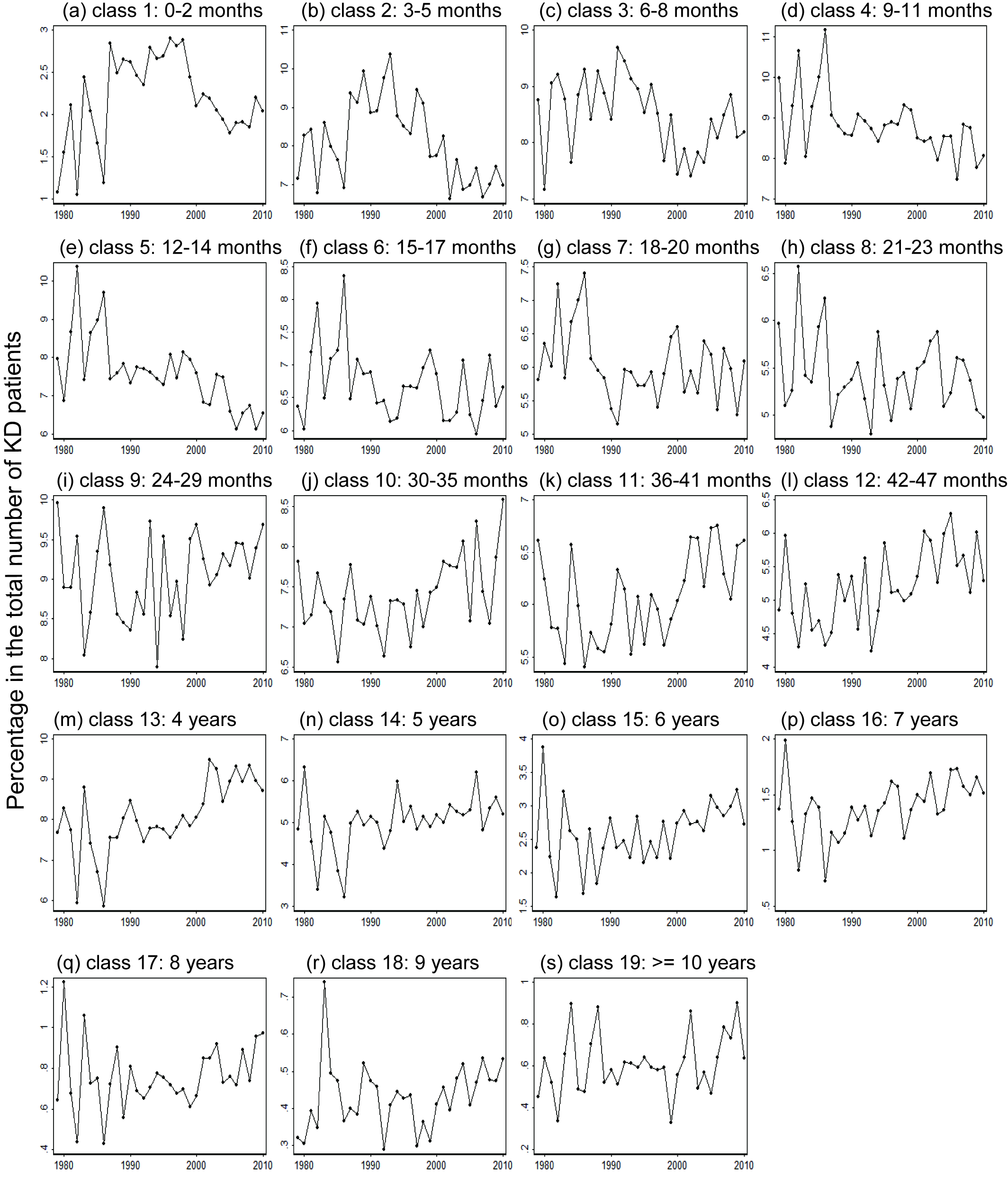

Supplement: Figure S4 — Percentage of KD patients in 19 age classes. (TIF) [file pone.0067934.s004.tif]

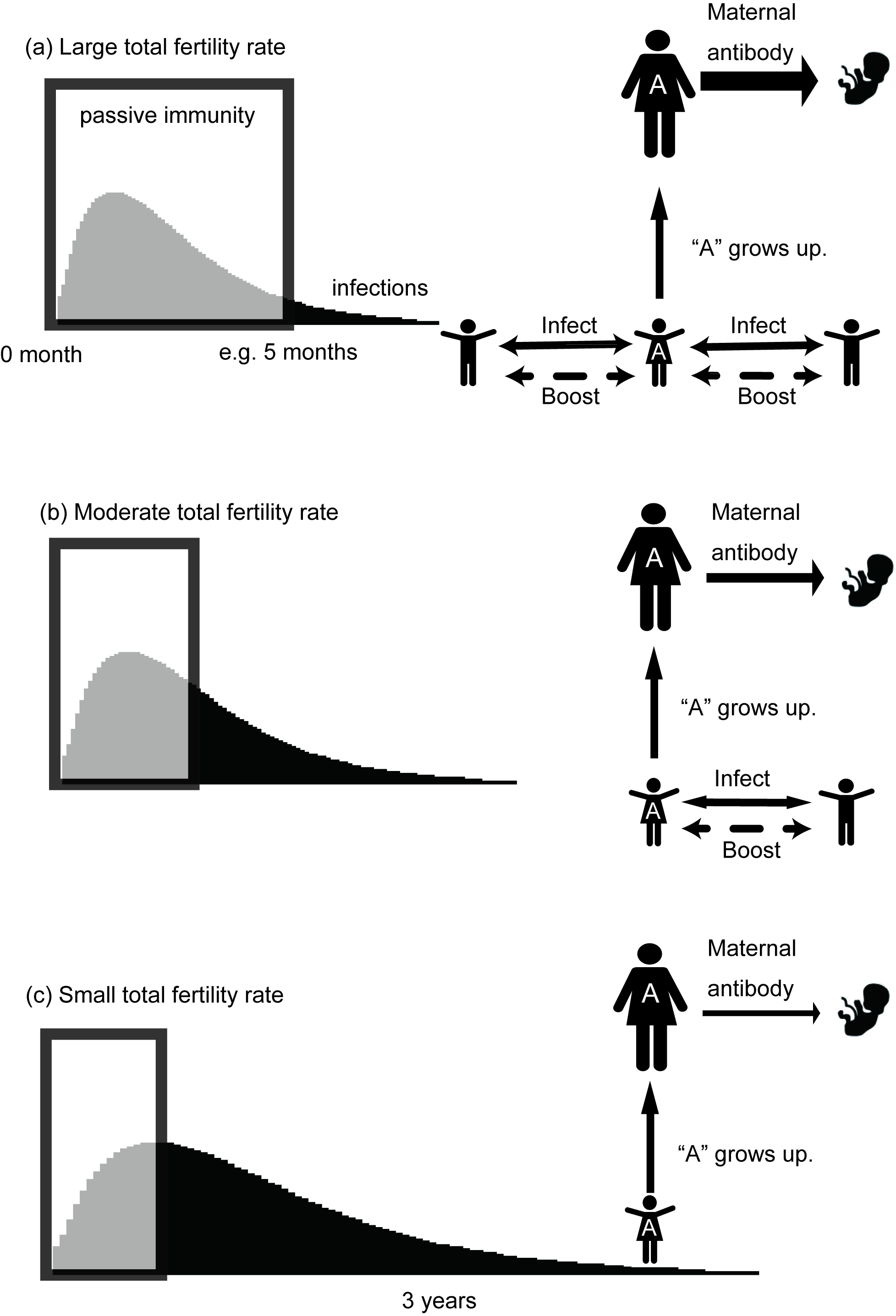

Supplement: Figure S5 — Hypothesis: decreasing total fertility rate may reduce the titre of maternal antibody, thereby increasing the incidence of KD. (TIF) [file pone.0067934.s005.tif]

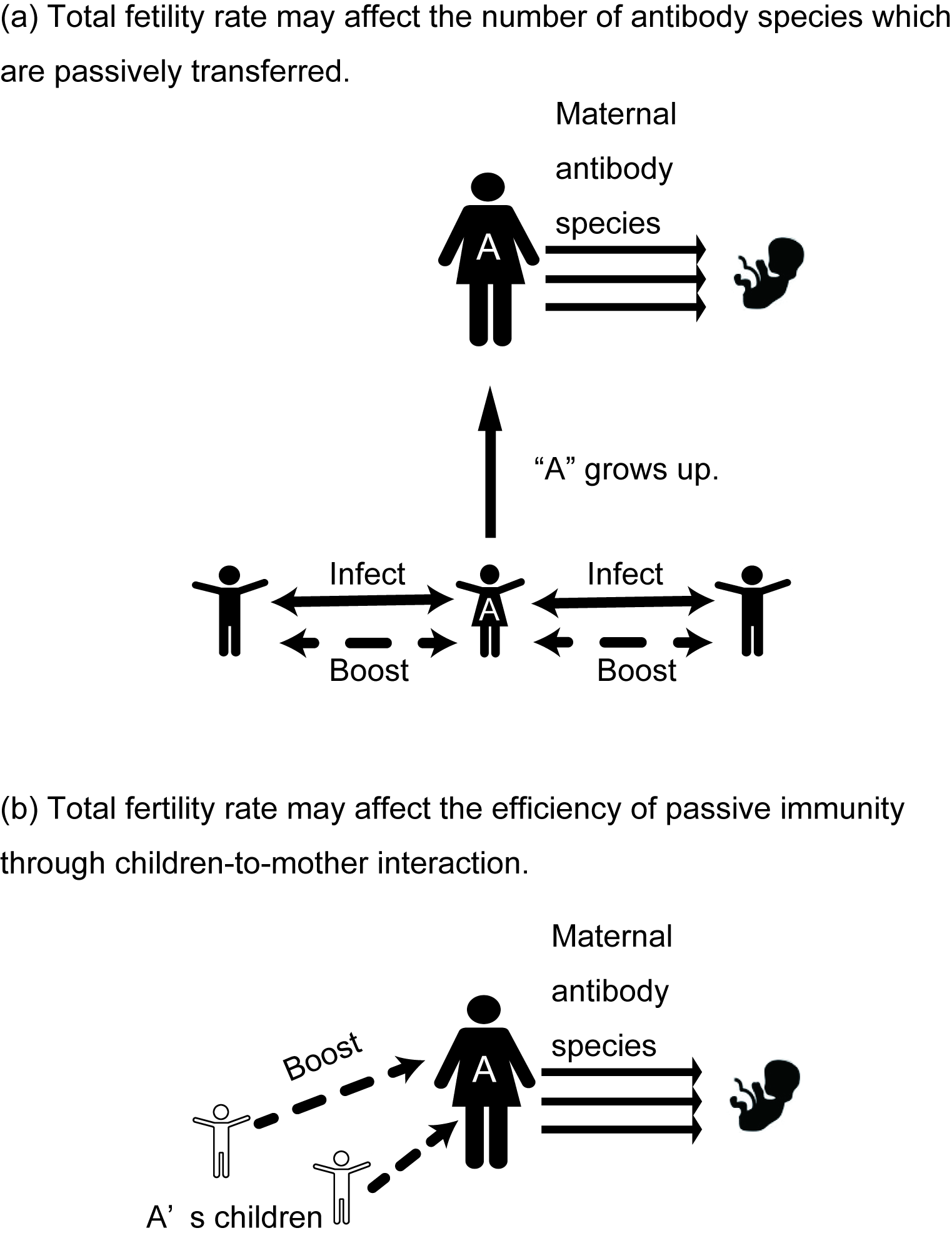

Supplement: Figure S6 — Alternative hypotheses. (a) Total fetility rate may affect the number of antibody species which are passively transferred. (b) Total fertility rate may affect the efficiency of passive immunity through children-to-mother interaction. (TIF) [file pone.0067934.s006.tif]
