# Supplementary material for: Decreasing Fertility Rate Correlates with the Chronological Increase and Geographical Variation in Incidence of Kawasaki Disease in Japan
Source: PLoS One. 2013 Jul 8;8(7):e67934. doi: 10.1371/journal.pone.0067934 (PMC3704585; doi:10.1371/journal.pone.0067934)
Supplement: Table S1 — Regression analyses from Table 2 (main text) was applied to non-normalized incidence of KD over 0–4 year old population (n = 47). (DOC) [file pone.0067934.s007.doc]

**Table S1.** Regression analyses from Table 2 (main text) was applied to non-normalized incidence of KD over 0–4 year old population (n=47).

| **Variables** |  |
| --- | --- |
| **Univariate regression** |  |
| Mean temperature  R^2^ | -1.2 (P=0.256)  0.029 |
| Rainfall | -0.042 (P=0.641) |
| R^2^ | 0.0049 |
| Physician | 0.055 (P=0.477) |
| R^2^ | 0.011 |
| Population density | 0.0021 (P=0.352) |
| R^2^ | 0.019 |
| Aged population | -0.33 (P=0.727) |
| R^2^ | 0.0027 |
| Higher education  R^2^ | 3.5 (P=0.109)  0.056 |
| TFR | -67 (P<0.001) |
| R^2^ | 0.24 |
| **Conventional multivariate regression*** |  |
| TFR | -67 (P<0.001) |
| R^2^ | 0.24 |
| **Spatial multivariate regression*** |  |
| TFR | -63 (P<0.001) |
| *ρ* | 0.011 |
| R^2^ | 0.25 |

*: Only TFR remained as the statistically significant contributor to the multivariate model in both conventional and spatial regressions.
