# Supplementary material for: Decreasing Fertility Rate Correlates with the Chronological Increase and Geographical Variation in Incidence of Kawasaki Disease in Japan
Source: PLoS One. 2013 Jul 8;8(7):e67934. doi: 10.1371/journal.pone.0067934 (PMC3704585; doi:10.1371/journal.pone.0067934)
Supplement: Table S2 — Regressive analyses from Table 3 (main text) was applied to non-normalized mean age of KD patients (n = 47). (DOC) [file pone.0067934.s008.doc]

**Table S2. Regressive analyses from Table 3 (main text) was applied to non-normalized mean age of KD patients (n=47).**

|  | **Crude mean age** | **Adjusted mean age** |
| --- | --- | --- |
| **Univariate regression** |  |  |
| Mean temperature | -0.011 (P=0.098) | -0.0051 (P=0.174) |
| R^2^ | 0.060 | 0.041 |
| Rainfall | -0.00015 (P=0.785) | -0.00015 (P=0.635) |
| R^2^ | 0.0017 | 0.0050 |
| Physician | -0.00037 (P=0.424) | -0.00014 (P=0.603) |
| R^2^ | 0.014 | 0.0060 |
| Population density | 1.8*10^-6^ (P=0.894) | 0.000016 (P=0.036) |
| R^2^ | 0.0004 | 0.094 |
| Aged population | 0.0012 (P=0.829) | -0.0047 (P=0.144) |
| R^2^ | 0.0011 | 0.047 |
| Higher education  R^2^ | 0.0059 (P=0.649)  0.0047 | -0.00094 (P=0.903)  0.0003 |
| TFR | -0.35 (P=0.002) | -0.24 (P<0.001) |
| R^2^ | 0.19 | 0.25 |
| **Conventional multivariate regression*** |  |  |
| TFR | -0.35 (P=0.002) | -0.24 (P<0.001) |
| R^2^ | 0.19 | 0.25 |
| **Spatial multivariate regression**^†^ |  |  |
| Population density | -0.000029 (P=0.025) |  |
| TFR | -0.45 (P<0.001) | -0.20 (P<0.001) |
| *ρ* | 0.0086 | 0.0092 |
| R^2^ | 0.36 | 0.39 |

*: Only TFR remained as the statistically significant contributor to the multivariate model in conventional regressions both for crude and adjusted mean patient ages. †: In spatial regression, population density and TFR remained as significant contributors to crude mean age, while only TFR remained as the significant contributor to adjusted mean age.
