# Supplementary material for: Decreasing Fertility Rate Correlates with the Chronological Increase and Geographical Variation in Incidence of Kawasaki Disease in Japan
Source: PLoS One. 2013 Jul 8;8(7):e67934. doi: 10.1371/journal.pone.0067934 (PMC3704585; doi:10.1371/journal.pone.0067934)
Supplement: Table S3 — Regression analyses from Table 4 (main text) was applied to non-normalized KD incidence. (DOC) [file pone.0067934.s009.doc]

**Table S3. Regression analyses from Table 4 (main text) was applied to non-normalized KD** incidence.

|  |  |  | **Incidence** |
| --- | --- | --- | --- |
| **Univariate analysis, 2000-2010** | | | |
|  | ***G*** | ***W*** | **(n=517)** |
| Mean temperature | 20 | 1 | -0.77 (*P*=0.661) |
| R^2^ |  |  | 0.0061 |
| Rainfall | 20 | 1 | -0.20 (*P*=0.083) |
| R^2^ |  |  | 0.0091 |
| Physician | 19 | 1 | 1.1 (*P*<0.001) |
| R^2^ |  |  | 0.22 |
| Population density | 23 | 1 | 0.0071 (*P*=0.108) |
| R^2^ |  |  | 0.015 |
| Aged population | 14 | 1 | 14 (*P*<0.001) |
| R^2^ |  |  | 0.075 |
| Higher education | 13 | 1 | 19 (*P*<0.001) |
| R^2^ |  |  | 0.063 |
| TFR | 15 | 2 | -238 (*P*<0.001) |
| R^2^ |  |  | 0.37 |
| **Multivariate analysis, 2000-2010** | | | |
|  | ***G*** | ***W*** | **(n=517)** |
| Aged population | 14 | 1 | 5.8 (P<0.001) |
| TFR | 15 | 2 | -179 (P<0.001) |
| R^2^ |  |  | 0.42 |
| **Multivariate analysis, 1979-2010** | | | |
|  | ***G*** | ***W*** | **(n=893)** |
| Aged population | 14 | 1 | 9.0 (P<0.001) |
| TFR | 15 | 2 | -155 (P<0.001) |
| R^2^ |  |  | 0.58 |
