# Supplementary material for: Decreasing Fertility Rate Correlates with the Chronological Increase and Geographical Variation in Incidence of Kawasaki Disease in Japan
Source: PLoS One. 2013 Jul 8;8(7):e67934. doi: 10.1371/journal.pone.0067934 (PMC3704585; doi:10.1371/journal.pone.0067934)
Supplement: Table S4 — Regression analyses from Table 5 (main text) was applied to non-normalized mean patient age. (DOC) [file pone.0067934.s010.doc]

**Table S4. Regression analyses from Table 5 (main text) was applied to non-normalized mean patient** age.

|  |  |  | **Crude mean age** |  |  | **Adjusted mean age** |
| --- | --- | --- | --- | --- | --- | --- |
| **Univariate analysis, 2000-2010** | | | | | | |
|  | ***G*** | ***W*** | **(n=517)** | ***G*** | ***W*** | **(n=517)** |
| Mean temperature | 20 | 1 | -0.022 (*P*<0.001) | 14 | 1 | -0.013 (P=0.034) |
| R^2^ |  |  | 0.057 |  |  | 0.014 |
| Rainfall | 19 | 1 | -0.0011 (*P*=0.034) | 6 | 2 | -0.0011 (P=0.014) |
| R^2^ |  |  | 0.014 |  |  | 0.017 |
| Physician | 2 | 1 | -0.00087 (*P*=0.034) | 2 | 1 | -0.00098 (P=0.010) |
| R^2^ |  |  | 0.024 |  |  | 0.022 |
| Population density | 1 | 1 | 4.9×10^-6^ (*P*=0.734) | 1 | 1 | 0.000036 (P=0.003) |
| R^2^ |  |  | 0.0006 |  |  | 0.034 |
| Aged population | 22 | 1 | -0.0032 (*P*=0.642) | 7 | 1 | -0.013 (P<0.001) |
| R^2^ |  |  | 0.0033 |  |  | 0.048 |
| Higher education | 11 | 1 | 0.024 (P=0.013) | 2 | 1 | -0.013 (P=0.259) |
| R^2^ |  |  | 0.020 |  |  | 0.0026 |
| TFR | 22 | 1 | -0.37 (*P*<0.001) | 20 | 1 | -0.36 (P<0.001) |
| R^2^ |  |  | 0.061 |  |  | 0.059 |
| **Multivariate analysis, 2000-2010** | | | | | | |
|  | ***G*** | ***W*** | **(n=517)** | ***G*** | ***W*** | **(n=517)** |
| Physician | 2 | 1 | -0.0017 (P<0.001) | 2 | 1 | -0.0013 (P<0.001) |
| Higher education | 11 | 1 | 0.020 (P=0.014) |  |  |  |
| TFR | 22 | 1 | -0.43 (P<0.001) | 20 | 1 | -0.45 (P<0.001) |
| R^2^ |  |  | 0.12 |  |  | 0.10 |
| **Multivariate analysis, 1979-2010** | | | | | | |
|  | ***G*** | ***W*** | **(n=611)** | ***G*** | ***W*** | **(n=705)** |
| Physician | 2 | 1 | -0.0015 (P<0.001) | 2 | 1 | -0.0013 (P<0.001) |
| Higher education | 11 | 1 | 0.020 (P=0.007) |  |  |  |
| TFR | 22 | 1 | -0.55 (P<0.001) | 20 | 1 | -0.48 (P<0.001) |
| R^2^ |  |  | 0.16 |  |  | 0.12 |
