# Supplementary material for: Decreasing Fertility Rate Correlates with the Chronological Increase and Geographical Variation in Incidence of Kawasaki Disease in Japan
Source: PLoS One. 2013 Jul 8;8(7):e67934. doi: 10.1371/journal.pone.0067934 (PMC3704585; doi:10.1371/journal.pone.0067934)
Supplement: Text S1 — Definition of Kawasaki disease. (DOC) [file pone.0067934.s011.doc]

**Text S1. Definition of Kawasaki disease.**

**Revision 3**

A case that fulfills five or more of the following criteria is diagnosed as Kawasaki disease:

- A fever of unknown cause persisting at least 5 days.
- Changes in extremities:

Acute: erythema of palms, soles; edema of hands, feet.

Recovery: peeling from periungual area.

- Polymorphous exanthem without blister or crust (this often appears on the trunk).
- Bilateral bulbar conjunctival injection (which could be transient).
- Changes in lips and oral cavity: cracking lips, erythema, strawberry tongue, diffuse injection of oral and pharyngeal mucosa.
- Non-purulent cervical lymphadenophathy during acute phase (which could be transient).

**Revision 4**

A case that fulfills five or more of the following criteria is diagnosed as Kawasaki disease:

- A fever persisting at least 5 days.
- Changes in extremities:

Acute: erythema of palms, soles; edema of hands, feet.

Recovery: peeling from the tip of finger(s).

- Polymorphous exanthem.
- Bilateral bulbar conjunctival injection.
- Changes in lips and oral cavity: cracking lips, erythema, strawberry tongue, diffuse injection of oral and pharyngeal mucosa.
- Non-purulent cervical lymphadenophathy during acute phase.

Even if only four out of the six criteria are fulfilled, the diagnosis of KD is made if coronary aneurysm (including dilatation) is detected by cardiac ultrasonography or angiogram and if other diseases are excluded.

**Revision 5**

The first item of revision 4 was modified into “a fever persisting at least 5 days (or a fever which subsides within 5 days due to treatment).”

**References**

1. Committee for Kawasaki disease (1978) Guideline for diagnosis of muco-cutaneous lymphnode syndrome (MCLS), revision 3. Tokyo: Ministry of Health Labour and Welfare.

2. Committee for Kawasaki disease (1984) Guideline for diagnosis of Kawasaki disease (Muco-cutaneous Lymphnode Syndrome), revision 4. Tokyo: Ministry of Health Labour and Welfare.

3. Committee for Kawasaki disease (2002) Guideline for diagnosis of Kawasaki disease (Muco-cutaneous Lymphnode Syndrome), revision 5. Tokyo: Ministry of Health Labour and Welfare.
