# Supplementary material for: Decreasing Fertility Rate Correlates with the Chronological Increase and Geographical Variation in Incidence of Kawasaki Disease in Japan
Source: PLoS One. 2013 Jul 8;8(7):e67934. doi: 10.1371/journal.pone.0067934 (PMC3704585; doi:10.1371/journal.pone.0067934)
Supplement: Text S2 — Hypotheses to explain increasing incidence of KD based upon decreasing total fertility rate. (DOC) [file pone.0067934.s012.doc]

**Text S2. Hypotheses to explain increasing incidence of KD based upon decreasing total fertility rate.**

The “inefficient passive immunity” hypothesis may explain the finding that decreases in the TFR were associated with increases in KD with a time lag of 15 years. Under a very high TFR, a girl would have been frequently infected and boosted by her siblings and friends of the same generation, and consequently acquired a high antibody titer against the pathogen that causes KD (Supplementary Figure S5 (a)). When the girl is older and has her child, she would transfer a large amount of maternal antibody to her child. As a result, the period of effective passive immunity in her baby would be long (e.g., 5 months); therefore, KD incidence in children 0–5 months of age would be very low. In addition, during this period, an infant of this age class would frequently encounter the KD pathogen and could seroconvert without manifesting severe symptoms. This assumption is supported by numerous reports that vaccination of infants in the presence of maternal antibodies leads to seroconversion in many vaccine recipients .

As the TFR decreased, the frequency of being infected or boosted by a girl’s siblings and friends decreased, and the period of passive immunity in her future baby is shortened (Supplementary Figure S5 (b)). As a result, the incidence in infants, aged 0–5 months, increases. Meanwhile, the decrease in the number of siblings also leads to a decrease in the hazard of contracting the aetiologic agent, elevating the mean age of infection (Supplementary Figure S5 (c)). Therefore, the incidence in the older age classes (≥3 years) finally increases.

There are alternative hypotheses that could explain the positive link between the TFR and KD incidence. For example, it was suggested that KD may be caused by multiple agents . Under a high TFR and the consequent strong force of infection, a girl would be infected and seroconverted to multiple KD pathogens and would transfer a larger number of antibody species to her future babies (Supplementary Figure S6 (a)). As the TFR (and the hazard of contracting the aetiologic agent) decreases, the number of antibodies transferred to the fetus decreases, thereby presenting a state of inefficient passive immunity.

Another alternative hypothesis is that passive maternal immunity is dependent on a woman’s interactions with her children (Supplementary Figure S6 (b)).

References to this Supplementary File

1. Osei-Kwasi M, Afari EA, Mimura K, Obeng-Ansah I, Ampofo WK, et al. (1995) Randomized, controlled trial of trivalent oral poliovirus vaccine (Sabin) starting at birth in Ghana. Bull World Health Organ 73: 41-46.

2. Kurubi J, Vince J, Ripa P, Tefuarani N, Riddell M, et al. (2009) Immune response to measles vaccine in 6 month old infants in Papua New Guinea. Trop Med Int Health 14: 167-173.

3. Kim D, Huey D, Oglesbee M, Niewiesk S (2011) Insights into the regulatory mechanism controlling the inhibition of vaccine-induced seroconversion by maternal antibodies. Blood 117: 6143-6151.

4. Pitzer VE, Burgner D, Viboud C, Simonsen L, Andreasen V, et al. (2012) Modelling seasonal variations in the age and incidence of Kawasaki disease to explore possible infectious aetiologies. Proceedings Biological sciences / The Royal Society 279: 2736-2743.
